# Rapid Embolic Event Chain: An Unusual Presentation of Marantic Endocarditis

**DOI:** 10.7759/cureus.83667

**Published:** 2025-05-07

**Authors:** Camron Costa, Fazal Dalal, Olajide Buhari, Jerry Fan, Steven Costa

**Affiliations:** 1 Internal Medicine, Baylor Scott & White Medical Center - Temple, Temple, USA; 2 Cardiology, Baylor Scott & White Medical Center - Temple, Temple, USA

**Keywords:** critical limb ischemia, embolic phenomenon, marantic endocarditis, nonbacterial thrombotic endocarditis (ntbe), stemi

## Abstract

Nonbacterial thrombotic endocarditis (NBTE), formerly known as marantic endocarditis, is characterized by sterile, noninfectious vegetations typically associated with hypercoagulable states, most commonly malignancies. Despite its noninfectious nature, NBTE can lead to serious complications similar to those seen in infectious endocarditis, including stroke, venous thromboembolism, splenic or hepatic infarcts, and acute intestinal ischemia. We present an unusual case involving a cascade of rapid embolic events in a patient newly diagnosed with metastatic lung cancer. The patient developed critical limb ischemia requiring bilateral below-knee amputation, with an echocardiogram revealing mitral valve vegetations. Postoperatively, the patient suffered an acute stroke and acute coronary syndrome, ultimately resulting in death. This case underscores the importance of early recognition and intervention in NBTE to prevent severe thromboembolic complications.

## Introduction

Nonbacterial thrombotic endocarditis (NBTE) is a rare condition first described in 1888 as an autopsy finding, characterized by sterile vegetations attached to cardiac valves [1]. It accounts for an estimated 0.9-1.6% of all endocarditis cases [1]. Approximately 75% of NBTE cases are associated with malignancy, while the remaining cases arise from noncancerous causes [2]. Although the exact trigger for NBTE remains unclear, it is thought to result from a combination of endothelial damage and hypercoagulability, leading to fibrin deposition and platelet aggregation on the heart valves [3]. A key distinction from infective endocarditis is that the vegetations in NBTE are more friable and more prone to systemic embolization [4]. While NBTE can affect any valve, it most commonly involves the mitral and aortic valves [5]. In contrast to infective endocarditis, NBTE-related immune complexes can affect both normal and abnormal valves, as well as the chordae tendineae and endocardium [5].

Although NBTE can occur across all patient populations, a study by Alhuarrat et al. found that it predominantly affects females and Caucasian individuals [2]. Outcomes vary based on underlying etiology, but the all-cause in-hospital mortality rate for NBTE admissions is approximately 36%, with even higher rates observed in cancer-associated cases [2]. Social determinants of health may also significantly influence outcomes in this population, as barriers to timely healthcare access can contribute to increased mortality. While data on the impact of social factors is limited, partly due to the rarity and frequent underdiagnosis of NBTE, it is plausible that certain cohorts experience worse outcomes due to these disparities. Unfortunately, NBTE is often diagnosed late, commonly post-mortem. We report a case of an atypical NBTE presentation marked by rapid clinical deterioration, beginning with a late diagnosis of malignancy and followed by a cascade of embolic events that ultimately led to the patient’s death.

## Case presentation

A 66-year-old man was transferred from an outside hospital in Australia after experiencing acute left leg pain and paresthesia in his toes. On examination, his bilateral lower extremities were cold to the touch; the left leg appeared more mottled, with no palpable pulses and mild erythema. He reported intermittent night sweats and weight loss over the past few months. He also had a significant smoking history, having smoked a pack of cigarettes per day for many years. Clinical findings raised suspicion for acute limb ischemia, prompting an emergency thrombectomy.

Further evaluation with CT imaging revealed widespread metastatic disease involving the liver, spine, and peritoneum. At that time, the patient had no known diagnosis of active malignancy. Despite undergoing thrombectomy, he continued to experience leg pain and ischemia. Additional imaging showed a new vascular occlusion, and a repeat thrombectomy was unsuccessful.

**Figure 1:**
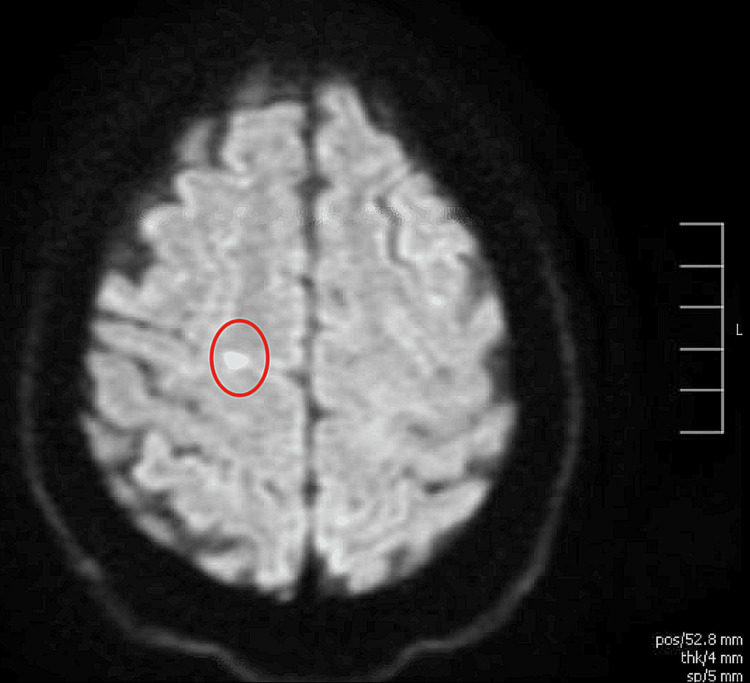
MRI of the brain showing an acute infarct in the precentral gyrus

**Figure 2:**
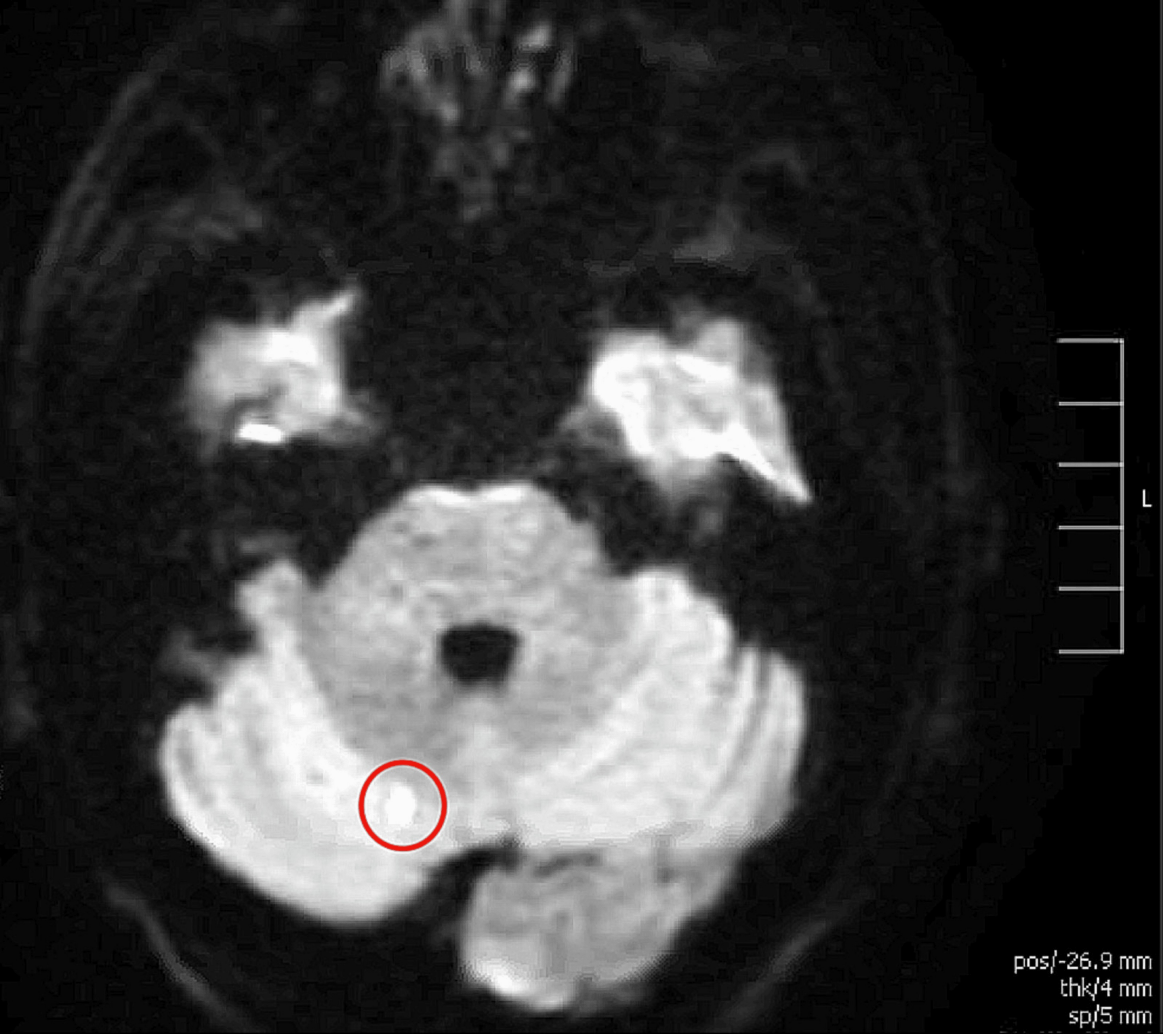
MRI of the brain showing an acute infarct in the right cerebellum

The patient subsequently developed acute stroke-like symptoms, primarily left upper extremity weakness and aphasia. MRI of the brain revealed acute infarcts in the precentral gyrus and right cerebellum (Figure 1, Figure 2).

**Figure 3:**
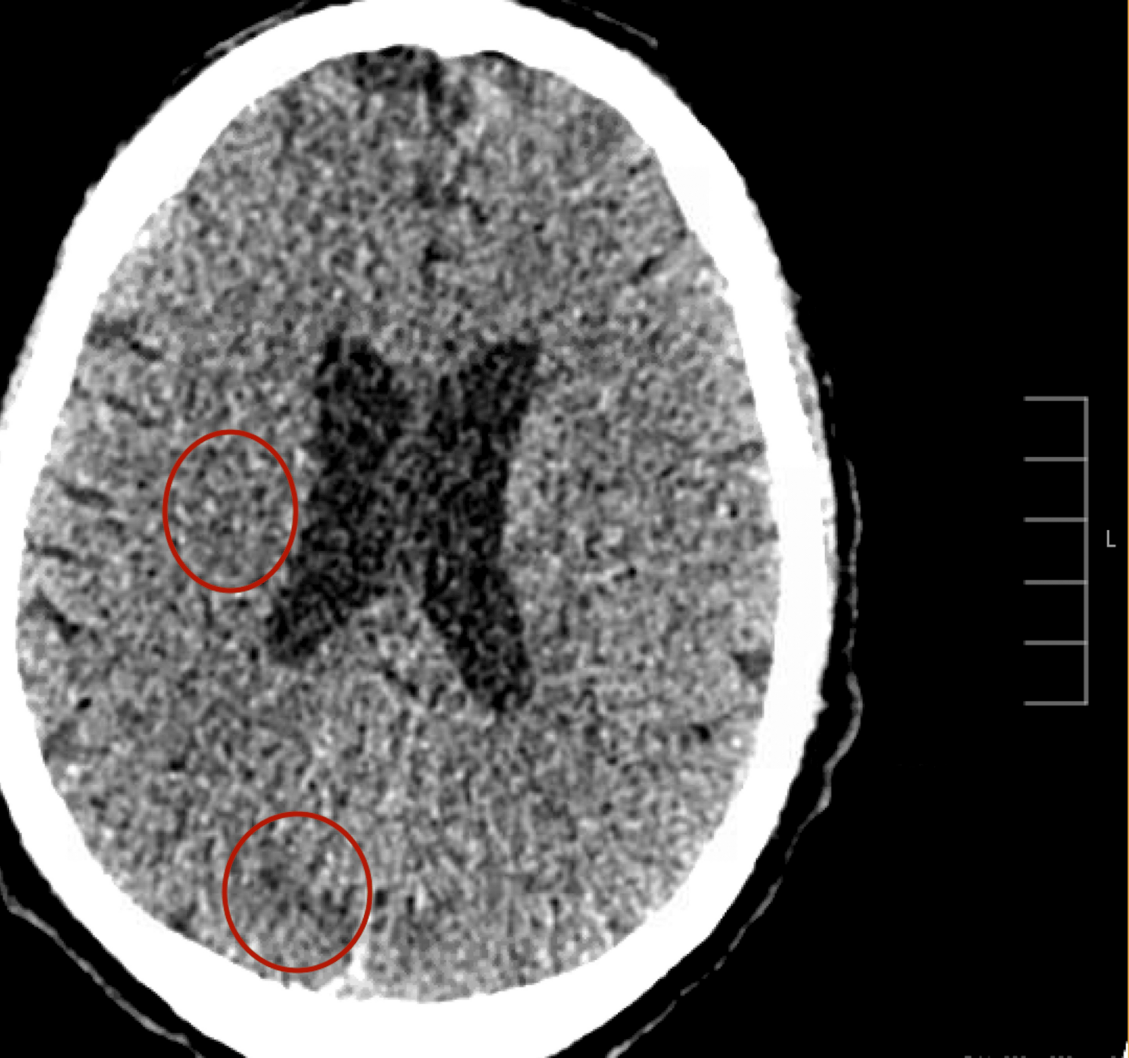
CT head showing acute infarct and early ischemic changes in the right medial parietal and occipital lobes

The patient was subsequently transferred to our institution in the United States. Upon arrival, vascular surgery was consulted, and the team offered conservative management with anticoagulation versus palliative amputations. The patient ultimately opted for surgical intervention, and the decision was made to proceed with palliative bilateral above-knee amputations due to the extent of the thromboembolic disease. Biopsy results from the outside hospital in Australia later confirmed poorly differentiated adenocarcinoma. Following the amputations, the patient’s condition continued to deteriorate. He experienced another stroke-like episode during the post-anesthesia recovery phase. A CT scan of the head revealed new acute infarcts in the right medial parietal region (Figure 3).

**Figure 4:**
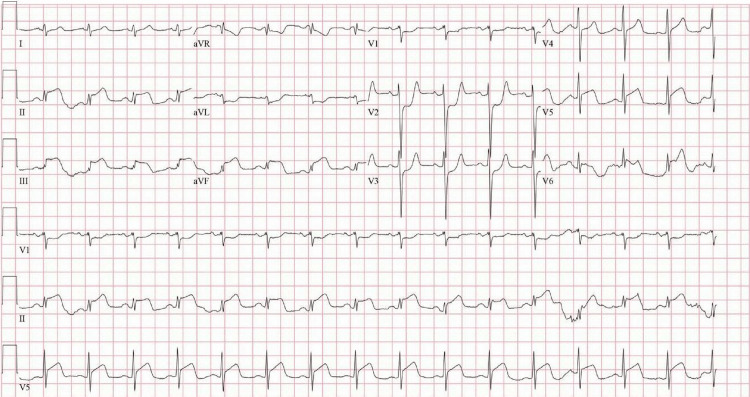
ECG showing acute inferolateral STEMI STEMI, ST-segment elevation myocardial infarction

**Figure 5:**
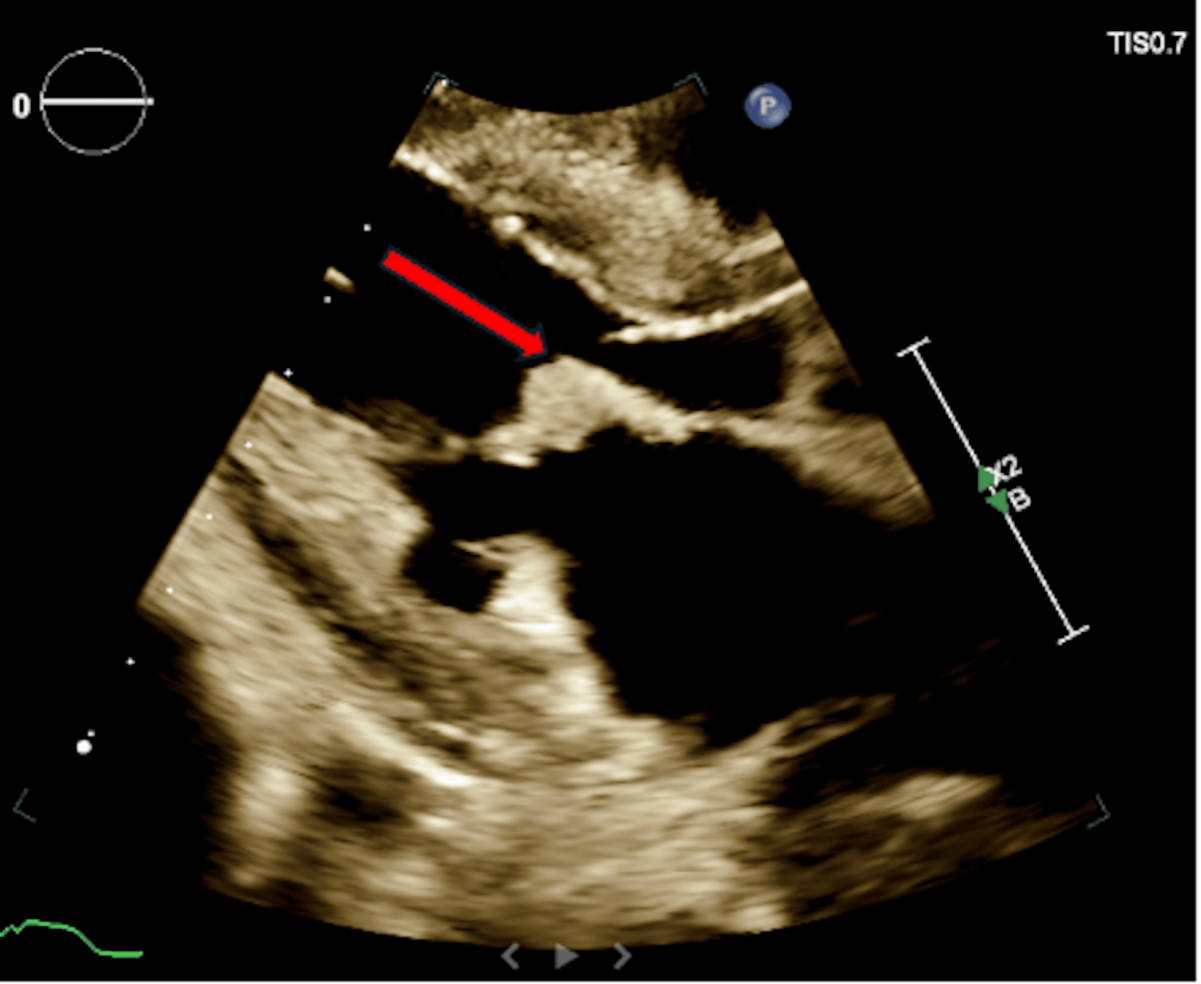
Parasternal long axis: thickening of the mitral leaflets with a mobile mass attached to the anterior leaflet of the mitral valve

**Figure 6:**
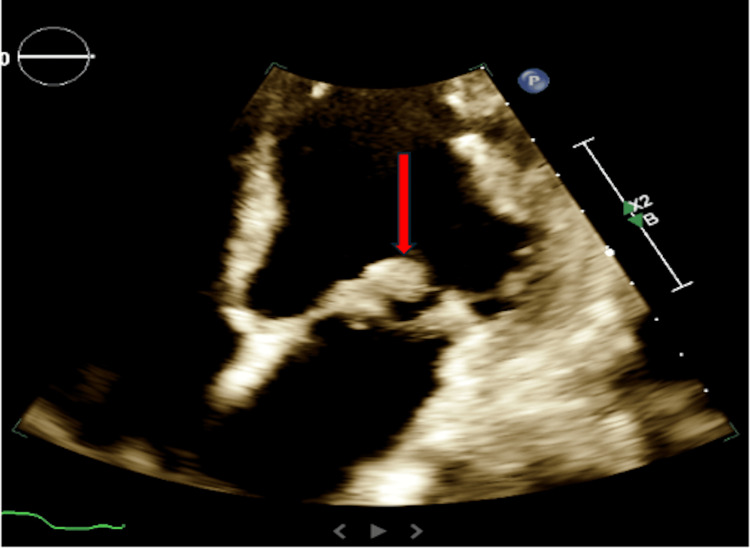
Apical 4-chamber: thickening of the mitral leaflets with a mobile mass attached to the anterior leaflet of the mitral valve

He continued to deteriorate and developed multi-organ failure, as evidenced by abnormal laboratory findings (Table 1). Vital signs during this period showed a blood pressure of 179/106 mmHg, a heart rate of 90 bpm, a respiratory rate of 34 breaths per minute, and an oxygen saturation of 100% on a 5 L nasal cannula. An ECG revealed an extensive inferolateral ST-segment elevation myocardial infarction (STEMI) (Figure 4). A transthoracic echocardiogram (TTE) demonstrated left ventricular systolic dysfunction with an ejection fraction of 35%, regional wall motion abnormalities consistent with coronary artery disease, and a 1.54 cm mobile mass attached to the anterior leaflet of the mitral valve, consistent with marantic endocarditis, suspected to be the source of embolization (Figure 5, Figure 6). A TTE was not initially ordered upon arrival, as the patient's primary complaint was leg pain related to known limb ischemia, and management was focused on that concern. However, his rapid decompensation and recurrent thrombotic events prompted evaluation for a cardioembolic source. He was not considered a candidate for revascularization due to the acute stroke and ongoing embolic events. As he was already on a heparin drip for critical limb ischemia, the STEMI was managed medically. Given his continued deterioration, the decision was made to transition him to comfort care. He passed away shortly thereafter.

**Table 1:** Lab values indicating multi-organ failure

Parameter	Observed value	Reference range
White blood cells	31.1 × 10³/uL	4.5-11.0 × 10³/uL
Hemoglobin	8.4 g/dL	14.0-18.0 g/dL
Creatinine	2.50 mg/dL	0.50-1.30 mg/dL
Potassium	5.4 mEq/L	3.5-5.3 mEq/L
Phosphorus	7.0 mg/dL	2.4-4.2 mg/dL
Calcium	10.6 mg/dL	8.6-10.5 mg/dL
Alkaline phosphatase	335 U/L	34-130 U/L
Aspartate aminotransferase	223 U/L	1-40 U/L
Alanine aminotransferase	85 U/L	0-68 U/L
Troponin I	1.98 ng/mL	0.00-0.09 ng/mL
B-type natriuretic peptide	279 pg/mL	0-100 pg/mL
Lactic acid	8.6 mmol/L	0.5-2.0 mmol/L

## Discussion

NBTE can occur in a variety of clinical scenarios. While it is most commonly associated with underlying malignancies, it has also been reported in autoimmune conditions such as systemic lupus erythematosus [6]. In the clinical case presented, the patient was newly diagnosed with primary lung adenocarcinoma with widespread metastatic disease. Blood cultures were not obtained due to the rapid progression of the disease, but the patient remained afebrile throughout the hospitalization. The rapid onset of multiple thromboembolic events - acute limb ischemia, stroke, and acute coronary syndrome - was unusual and made the diagnosis of NBTE more likely [7]. Cardioembolic phenomena are often mistaken for isolated events; however, the occurrence of multiple embolic events should raise suspicion for NBTE, particularly in the context of hypercoagulable or autoimmune conditions [7]. NBTE frequently presents insidiously and is often diagnosed in the later stages of the disease. Unfortunately, it tends to progress rapidly in these patient populations, resulting in poor outcomes and often death. This highlights the critical importance of early detection and management.

When diagnosed early, the management of NBTE largely depends on addressing the underlying cause. Early echocardiography is essential for identifying NBTE, informing treatment strategies, and potentially reducing the risk of complications. In patients with hypercoagulable states, anticoagulation therapy is a cornerstone of management [8]. In cancer patients, the prompt initiation of chemotherapy can help control the primary disease and reduce thromboembolic risk [8]. Although surgical valve replacement is not typically indicated, it may be considered in refractory cases [8]. Ultimately, clinical judgment and timely diagnosis are key to improving survival in patients with NBTE.

Research suggests that females may have a higher prevalence of NBTE compared to males, with approximately 62% of cases occurring in women [9]. While malignancy remains the most common underlying cause, autoimmune and connective tissue disorders are also strongly associated with NBTE, particularly in female patients [6]. The prognosis for NBTE is generally poor, especially in cases related to malignancy [10]. In such instances, NBTE often presents in advanced cancer stages, where the disease has already metastasized extensively. Patients diagnosed with marantic endocarditis frequently have widespread metastatic disease, limiting chemotherapy options. As a result, the mortality rate in this patient population is extremely high, approximately 80% [11]. Given the complexity of these cases, a multidisciplinary approach is crucial to facilitate shared decision-making and ensure the best possible care.

## Conclusions

This case highlights the importance of early recognition and intervention in patients with NBTE, particularly when it is associated with malignancy. The rapid progression of thromboembolic events in this patient, who was newly diagnosed with lung cancer and widespread metastatic disease, underscores the critical need for timely diagnosis, early imaging, and preventive management strategies. Higher suspicion of this phenomenon should arise upon the first instance of a recurrent thrombotic event, as it may allow for earlier diagnosis through imaging. Despite the challenges in treating NBTE, especially in the context of advanced cancer, prompt identification and appropriate management, such as anticoagulation therapy and addressing the underlying malignancy, are essential to mitigating further thrombotic complications. Unfortunately, the poor prognosis associated with advanced cancer and NBTE, as demonstrated in this case, requires a comprehensive and multidisciplinary approach to care, focusing on both medical management and palliative support. It is possible that NBTE may accelerate mortality in this patient population, despite timely intervention. This case serves as a reminder that multiple embolic events should heighten suspicion for NBTE, enabling quicker intervention and potentially improving patient outcomes.

## References

[1] Rahouma M, Khairallah S, Dabsha A (2023). Lung cancer as a leading cause among paraneoplastic non-bacterial thrombotic endocarditis: a meta-analysis of individual patients’ data. Cancers (Basel).

[2] Alhuarrat MA, Garg V, Borkowski P (2024). Epidemiologic and clinical characteristics of marantic endocarditis: a systematic review and meta-analysis of 416 reports. Curr Probl Cardiol.

[3] Liu J, Frishman WH (2016). Nonbacterial thrombotic endocarditis: pathogenesis, diagnosis, and management. Cardiol Rev.

[4] Roldan CA, Sibbitt WL Jr, Qualls CR (2013). Libman-Sacks endocarditis and embolic cerebrovascular disease. JACC Cardiovasc Imaging.

[5] Deppisch LM, Fayemi AO (1976). Non-bacterial thrombotic endocarditis: clinicopathologic correlations. Am Heart J.

[6] Moyssakis I, Tektonidou MG, Vasilliou VA, Samarkos M, Votteas V, Moutsopoulos HM (2007). Libman-Sacks endocarditis in systemic lupus erythematosus: prevalence, associations, and evolution. Am J Med.

[7] Ferlan G, Fiorella A, De Pasquale C, Tunzi F (2010). Primary coronary embolism as an unusual manifestation of nonbacterial thrombotic endocarditis in a patient with gastric cancer. Cardiol Res Pract.

[8] Ahmed O, King NE, Qureshi MA (2025). Non-bacterial thrombotic endocarditis: a clinical and pathophysiological reappraisal. Eur Heart J.

[9] Quintero-Martinez JA, Hindy JR, El Zein S, Michelena HI, Nkomo VT, DeSimone DC, Baddour LM (2022). Contemporary demographics, diagnostics and outcomes in non-bacterial thrombotic endocarditis. Heart.

[10] Al Chalaby S, Makhija RR, Sharma AN, Majid M, Aman E, Venugopal S, Amsterdam EA (2022). Nonbacterial thrombotic endocarditis: presentation, pathophysiology, diagnosis and management. Rev Cardiovasc Med.

[11] Orfanelli T, Sultanik E, Shell R, Gibbon D (2016). Nonbacterial thrombotic endocarditis: a rare manifestation of gynecologic cancer. Gynecol Oncol Rep.

